# Metabolic Regulation, Oxygen Limitation and Heat Tolerance in a Subtidal Marine Gastropod Reveal the Complexity of Predicting Climate Change Vulnerability

**DOI:** 10.3389/fphys.2020.01106

**Published:** 2020-09-15

**Authors:** David J. Marshall, Christopher D. McQuaid

**Affiliations:** ^1^Environmental and Life Sciences, Faculty of Science, Universiti Brunei Darussalam, Bandar Seri Begawan, Brunei Darussalam; ^2^Department of Zoology and Entomology, Rhodes University, Grahamstown, South Africa

**Keywords:** metabolic depression, hypoxia, air exposure, snail, subtidal, thermal tolerance, climate change

## Abstract

Predictions for climate vulnerability of ectotherms have focused on performance-enhancing physiology, even though an organism’s energetic state can also be balanced by lowering resting maintenance costs. Adaptive metabolic depression (hypometabolism) enables animals to endure food scarcity, and physically extreme and variable environmental conditions. Hypometabolism is common in terrestrial and intertidal marine gastropod species, though this physiology and tolerance of environmental change are poorly understood in subtidal benthic gastropods. We investigated oxygen limitation tolerance, hypometabolism and thermal performance in the subtidal, tropical snail *Turritella bacillum*. Survival, cardiac activity and oxygen debt repayment were determined when oxygen uptake was limited by gill function impairment (air exposure) or exposure to hypoxic seawater. Thermal performance and tolerance were assessed from survival and cardiac performance when heated. The ability of snails to regulate metabolism during oxygen limitation was demonstrated by their tolerance of air exposure (>36 h) and hypoxia (>16 h), rhythmicity and reversibility of bradycardia, and inconsistent anaerobic compensation. Under acute heating, mean heart rate was temperature-insensitive in water and temperature-dependent in air. Converging or peaking of individual heart rates during heating suggest maximization of thermal performance at 38–39°C, whereas survival and heartbeat flatlining suggest an upper thermal limit exceeding 42°C. Snails survived 16 h in seawater at 38°C. Their metabolic regulation complies with the oxygen-limiting, sediment-burrowing lifestyle of the species. Although a tropical organism, the species’ thermal tolerance so far exceeds present habitat temperatures as to question its susceptibility to centennial climate warming. Our findings reveal the importance of knowing the metabolic regulatory capabilities and conserved physiological attributes of species used in climate vulnerability tests. Studies of ectotherm climate vulnerability that identify generalized trends based on physiologically similar animals may be misleading by missing information on physiological diversity.

## Introduction

Metabolic rate depression (hypometabolism) is seen in most metazoan groups, especially among animals that hibernate, estivate or undergo diapause (Storey and Storey, 1990; Guppy and Withers, 1999; Marshall et al., 2011). By reducing cellular energy demand, by lowering rates of membrane ion pumping and macromolecule synthesis, less energy is required to achieve a balanced state and energy reserves are conserved (Grieshaber et al., 1994; Hand and Hardewig, 1996; Guppy and Withers, 1999). A lowered cellular energy demand thus enables the endurance of reduced food intake when food is scarce or during behavioral isolation to avoid exposure to extreme and adverse conditions (Table 1). It is thus remarkable that a physiological capability that is crucial to surviving environmental uncertainty is missing from mainstream climate vulnerability contexts. Climate vulnerability models for ectotherms focus on the maximization of performance and energy intake relative to temperature, to the exclusion of processes that achieve energetic equilibrium by lowering resting metabolic demand (Brown et al., 2004; Seibel and Drazen, 2007; Sinclair et al., 2016; Huey and Kingsolver, 2019; but for alternatives, see Quévreux and Brose, 2019). To some extent this is understandable as the capability of hypometabolism is limited in many key animal groups, while in others (such as gastropods) its phylogenetic distribution is poorly known. Nevertheless, omitting this aspect of the energy equation challenges the views founded on performance maximization that tropical and marine ectotherms are likely to be especially threatened by climate warming (Deutsch et al., 2008, 2015; Huey et al., 2009; Pinsky et al., 2019).

**TABLE 1:** Behavioral and environmental drivers and hierarchical responses during marine gastropod hypometabolism.

	Behavioral	Environmental
	Hypometabolism controlled by the organism in response to a feeding limitation, sand burial or behavioral isolation (withdrawal into the shell)	Hypometabolism associated with internal hypoxia linked to abiotic environmental change, directly or through respiratory incapacity
Drivers	∙ Food limitation	∙ **Air exposure** of low intertidal or subtidal gastropods
	∙ Sand burial	
	∙ Predator avoidance	∙ **Hypoxic seawater exposure**
	∙ Prevent exposure to abiotic extremes (salinity, pH, metal pollution, etc)	∙ Thermal extreme exposure
Respiratory organ	∙ Lowered gas exchangeand O_2_ uptake is potentially reversible with a change in behavior	∙ Lowered gas exchange and O_2_uptake relates to external conditions or respiratory organ incapacity and is non-reversible while conditions persist
Systemic	∙ Modulation of cardiac function including transient or sustained bradycardia retains systemic functionality or supports cellular O_2_ demand	∙ Cardiac stress below the internal hypoxia tolerance threshold seen as episodes of tachycardia and/or acardia and cardiac arrest
	∙ Reduction of blood pO_2_ and pH and elevation of blood pCO_2_ (isolation)	∙ Reduction of blood pO_2_ and pH and elevation of blood pCO_2_ (air) or only reduction of blood pO_2_ (environmental hypoxia)
Cellular	∙ Metabolic downregulation seen by one or combinations of hemolymph reduced pO_2_, reduced pH and elevated pCO_2_	∙ Metabolic stressseen by one or combinations of hemolymph reduced pCO_2_, reduced pH and elevated pO_2_
	∙ Capable of voluntary temporary reversion of metabolic state to perform limited locomotion	∙ Reversion to a normal metabolic state that supports growth and reproduction requires normalization of environmental conditions and food uptake
	∙ Reversion to a normal metabolic state that supports growth and reproduction requires change of behavior and food uptake	

*Drivers tested in this study are indicated in bold. See Marshall and McQuaid (1991, 1992a, 1993, 2011) and Marshall et al. (2011).*

In gastropod mollusks, metabolic depression likely originated in early marine lineages along with predation avoidance behavior (Palmer, 1979), as withdrawal into the shell occludes respiratory gas exchange. This physiology is crucial to the unique radiation of gastropods in all of Earth’s domains (Webb, 2012). By enabling behavioral isolation, limiting environmental contact and reducing evaporative water loss from an organism, hypometabolism underpins the evolutionary transition of gastropods between marine and terrestrial ecosystems, and is a prerequisite for their life on land (Storey and Storey, 1990; Guppy and Withers, 1999; Strong et al., 2008; Webb, 2012). Although, hypometabolism facilitates estivation and prolonged air exposure in intertidal marine snails (sandy beaches, rocky shores, mudflats, and mangroves; Marshall and McQuaid, 2011; Marshall et al., 2011), little is known about its occurrence in benthic, subtidal gastropods (see Table 1), which often show significant tolerance of environmental hypoxia (Riedel et al., 2012). The primary environmental triggers of hypometabolism in marine gastropods are reduced oxygen uptake and reduced food intake. Oxygen uptake becomes limited when seawater oxygen tensions decline, and when gill function is impaired during air exposure in intertidal gastropods (Table 1). Oxygen limitation is behaviorally induced when snails retract into the shell to avoid unfavorable abiotic exposures (air, salinity, pollution or pH) or during sand burial (McMahon and Russell-Hunter, 1978; Brown and Da Silva, 1979; Little and Stirling, 1984; Kapper and Stickle, 1987; Liu et al., 1990; Marshall and McQuaid, 1993; Marshall et al., 2004, 2011; Marshall, 2009; Proum et al., 2017; Table 1). The induction and deactivation of hypometabolism in intertidal gastropods is rapid in order to offset temporal limitations on feeding, which can last from hours to months depending on the tide cycle and season (Marshall and McQuaid, 2011; Monaco et al., 2017). This differs fundamentally from hypometabolism of temperate terrestrial overwintering or diapause animals, which is usually intrinsically programmed and linked to seasonal developmental cycles (Storey, 2002; Storey and Storey, 2004).

Assessing metabolic downregulation often involves following the response of an organism to restricted oxygen uptake, which leads to cellular oxygen limitation and impeded ATP production (Table 1). Under such conditions, animals with limited capacity for metabolic depression are obliged to counter the shortfall in aerobically generated ATP through anaerobic production (Stickle et al., 1989; Liu et al., 1990). In animals capable of hypometabolism, anaerobic metabolism only occurs after the lowest depressed metabolic rate is reached (Grieshaber et al., 1994). Anaerobic metabolism can be determined from metabolite accumulation or inferred from an “oxygen debt” that accumulates under hypoxic conditions and is repaid when oxygen becomes available again (Hochachka, 1988). Oxygen debt repayment manifests as a respiratory or cardiac overshoot during recovery in normal oxygen seawater, and its magnitude essentially relates to the degree of anaerobic compensation (Brown and Wynberg, 1987; Hochachka, 1988; Marshall and McQuaid, 1993; Proum et al., 2017). In gastropods, features of cardiac functioning have also been used to assess hypometabolic state. Whereas harmonic bradycardia (regular beat-beat interval) indicates a stable lowered energetic state, intermittent tachycardia or acardia often signifies a respiratory stress response (Marshall and McQuaid, 1993, 1994, 2011; Marshall et al., 2004; Proum et al., 2017). Metabolic regulation is also implied from thermal insensitivity of the metabolic rate (Brown et al., 1978; Marshall and McQuaid, 1994, 2011; McMahon et al., 1995; Marshall et al., 2011; Verberk et al., 2016).

We propose that metabolic depression has critical implications for understanding species’ responses to long-term environmental change that are not considered in most predictions of how global warming will affect species (e.g., Brown et al., 2004; Huey and Kingsolver, 2019). To address this, we investigated metabolic depression and thermal tolerance in the tropical subtidal gastropod, *Turritella bacillum* (Turritellidae). By considering a largely unexplored model system, this study adds to understanding of the evolutionary and ecological origins of these traits and helps to evaluate the range of possible responses of gastropods to environmental change. *T. bacillum* is related to other cerithioidean species (Planaxidae, Potamididae, and Thiaridae) that have utilized metabolic depression and estivation to radiate in fringe environments (high-rocky shores, mangroves, and brackish and freshwater systems; Houbrick, 1988; Strong et al., 2008, 2011). Although *T. bacillum* lives in a thermally stable environment (25–31°C), as a species it dates to before the Plio-Pleistocene when temperatures were 2–3°C higher than present (Allmon, 2011; Das et al., 2018); the family dates back to the Cretaceous before the appreciably hotter Paleocene-Eocene Thermal Maximum (PETM, 5°C higher than today; Das et al., 2018; Ivany et al., 2018). Although they are key components of tropical marine ecosystems, information on the physiology of subtidal gastropods is missing from mainstream models for climate change vulnerability (Deutsch et al., 2008; Sunday et al., 2012,2014; Bennett et al., 2018; Pinsky et al., 2019). The broader objective here was to illustrate that physiological diversity can make predicting the consequences of climate change more complex than appreciated.

## Materials and Methods

### Snail Collection and Handling

**FIGURE 1:**
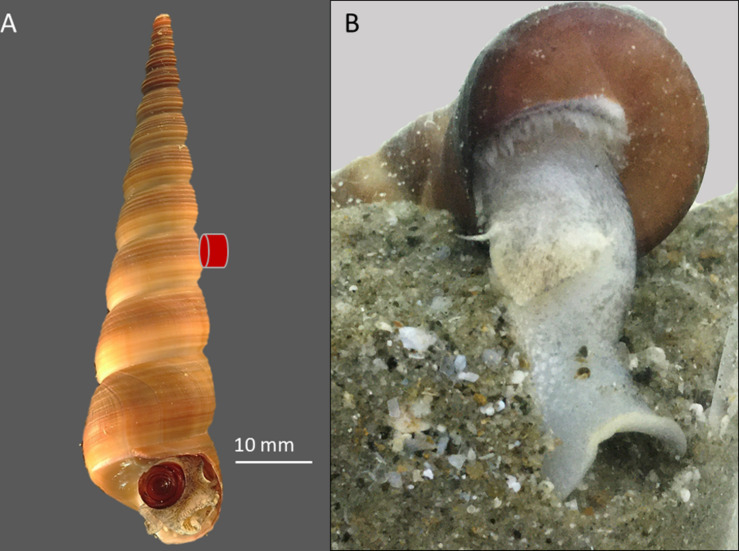
**(A)** *Turritella bacillum* photgraphed in air, showing retracted head-foot and operculum centrally in the aperture; this is also the assumed posture during sediment feeding. **(B)** Snail burrowing in a glass tank in the laboratory, showing extrusion and probing of the foot. The red symbol indicates the position of the IR sensor used to detect heartbeat.

*Turritella bacillum* (Kiener, 1843) is widespread in the South China Sea, having a southern equatorial limit in Malaysia and Indonesia and a northern distribution limit in Japan (Scarponi et al., 2018). It is a suspension-feeding subtidal gastropod that remains buried below the sediment surface when not searching for a mate (Figure 1; Allmon, 2011; Waite and Allmon, 2016). It occurs down to depths of ∼50 m and is reported to tolerate a range of seawater salinities (10–35 psu), temperatures (15–30°C), suspended solids (20 mg L^–1^) and dissolved oxygen (<5 mg L^–1^) (Trong et al., 2000; Kwan et al., 2018). The present range of temperatures in Brunei waters is 25–31°C (see Supplementary Figure 1; Lane, 2011; Johari and Akhir, 2019).

Snails for the present study were collected from the intertidal zone of a sandy beach at Pantai Tungku, Brunei Darussalam (4.974_N, 114.867_E), between December 2019 and February 2020. These snails had been dislodged from their subtidal habitat during monsoon high sea conditions. Early morning collections of >100 snails were made on several different days. Before experiments, the snails were kept for 1–3 d in glass tanks of recirculating water [60 cm (L) × 30 cm (W) × 40 cm (H); Eheim Professional pumps] at 27°C, 33 psu salinity and without the provision of planktonic food. Their survival in the laboratory was excellent, with only a two mortalities over 12 weeks. Snails provided with beach sediment rapidly buried themselves (Figure 1), remaining buried without contact with the overlying water for over 6 weeks. Snails not provided with sediment remained sedentary, though a few showed heave-crawling along the tank surface. When not moving, the head-foot and operculum were usually located centrally at the aperture (Figure 1). In some, the body was withdrawn into the shell, sometimes to near the third from last whorl. Although the small operculum does not completely cover the shell aperture, it minimizes contact of the body with the outside environment and prevents sediment intrusion when deeper in the shell. Retracted snails invariably re-emerged at the shell aperture within 30 min of exposure in air (24°C; benchtop temperature). Snails that were not sacrificed to determine tissue mass, were returned to the subtidal sea near to where they were collected.

### Oxygen Limitation: Tolerance and Metabolic Depression

We assessed survival and physiological responses of snails to restricted oxygen uptake through exposure to either air, which limits oxygen uptake at the gills, or hypoxic seawater (Table 1, Proum et al., 2017). Hypoxia leads only to lowered hemolymph PO_2_ while air exposure also causes elevated hypercapnia and potentially reduces hemolymph and cellular pH. In land snails, lowered hemolymph pH due to elevated hemolymph PCO_2_, when gas exchange is reduced during withdrawal into the shell, is the primary driver of metabolic downregulation (Barnhart and McMahon, 1988).

#### Survival of Oxygen Limitation

Tolerance of air exposure was determined for snails held in loose polythene bags in a Memmert Peltier-cooled (IPP400) incubator for 16 (*n* = 12), 24 (*n* = 12), 36 (*n* = 5) and 48 h (*n* = 6) at 27 ± 0.2°C and 95% R.H. (monitored using DS1923-F Hygrochron I-buttons). Survival of hypoxic seawater was determined for snails (*n* = 5) exposed for 16 h to 1.0–1.5 mg O_2_ L^–1^ (27 ± 0.2°C). Hypoxic conditions were created by bubbling mixtures of air and N_2_ gas into seawater (33 psu) in a 5-L beaker, held inside a Grant waterbath at 27 ± 0.2°C. The oxygen concentration (% Air Saturation) of the water was continuously monitored using a Witrox fiber-optic system (Loligo Systems, Denmark). In the present experiment, snails were retained in respiratory chambers (∼27 mL volume) inside the beaker, while the oxygen concentration of the water was lowered from ambient to 1.5 mg L^–1^ over 15 min. At 1.5 mg L^–1^ O_2_, the respiratory chambers were capped and left overnight for 16 h in the waterbath, allowing the snails to extract further oxygen from the chamber water. The oxygen concentrations of water in the chambers containing snails decreased by an average of 0.5 mg L^–1^ O_2_ during this period. After the 16 h period, survival was determined by the re-emergence of snails when exposed to air at 24°C (see above).

#### Determining Metabolic Regulation From Cardiac Activity

We monitored cardiac activity in snails exposed to air or hypoxic seawater. In air, cardiograms were collected over 16 h for four snails kept in loose polythene bags in a Memmert Peltier-cooled (IPP400) incubator (27 ± 0.2°C; 95% R.H.; DS1923-F Hygrochron I-buttons), and for hypoxic seawater exposure, cardiograms were collected for a single snail during the oxygen debt formation experiment (see below; 3 h, 2.02–1.3 mg L^–1^ O_2_). Heartbeats were detected with IR-sensors fitted to the outside of shells at the third from last whorl (Figure 1), and coupled to bridge amplifiers and a Powerlab system (ADInstruments, Australia; Marshall et al., 2011). Under normal circumstances, the cardiovascular system of gastropods functions to deliver oxygen to the cells (Marshall and McQuaid, 1992b). Unlike ciliary ventilation of the mantle cavity, perfusion [heart rate (HR) and stroke volume] in gastropods can be dynamically modulated (within minutes) in response to cellular oxygen demand, consequently it reflects demand as well as oxygen uptake and delivery (Marshall and McQuaid, 1992b, 2011; Marshall et al., 2004, 2011). From cardiogram traces and HR-time plots, we checked for features that typically indicate metabolic regulation, such as harmonic bradycardia (similarity of beat-to-beat variation) and the recoverability from this bradycardia. Capacity-limited, stressed animals do not recover their heartbeat rhythmicity, but instead show tachycardia interspersed with acardia (see section “Introduction”; Marshall and McQuaid, 1993, 2011).

#### Determining Metabolic Regulation From Oxygen-Debt Formation

The formation of an oxygen debt was assessed for individual snails by comparing the aquatic respiratory rate in normal seawater *before* and *after* exposure to air or hypoxic seawater. An overshoot in the recovery (*after*) respiratory rate was taken to indicate repayment of an oxygen debt formed under oxygen-limited conditions, with the degree of overshoot indicating the level of incapacity for total metabolic depression (see section “Introduction”). For the air exposure treatment, snails (*n* = 12) were held in air for 16 h, inside open respiratory chambers contained in open polythene bags [Memmert Peltier-cooled (IPP400) incubator; 27 ± 2°C (95% R.H)]. Two hypoxic seawater exposure treatments were used; snails were exposed either to 2.02–1.3 mg L^–1^ O_2_ for 3 h (treatment 1; *n* = 8) or to 1.5–1.0 mg L^–1^ O_2_ for 16 h (treatment 2; *n* = 4). The oxygen concentration of seawater contained in a 5-L beaker inside a Grant waterbath set to 27 ± 0.2°C was manually lowered (see above, “survival of oxygen limitation”). In treatment 1, the water was lowered from normoxia at 6.73 to 2.02 mg L^–1^ (over 10 min), kept stable at 2.02 ± mg L^–1^ (for 50 min) and then lowered further to 1.3 mg L^–1^ (for 100 min), giving a total hypoxia exposure of 160 min. Treatment 2 followed the same protocol as described under “survival of oxygen limitation,” giving an exposure of 1.5–1.0 mg L^–1^ O_2_ for 16 h.

In the *before* respiratory rate determinations, snails were left to settle in open respiratory chambers (25-27 mL water volume, excluding snails) in recirculating seawater for 30 min (27°C). The chamber lids were then secured underwater and the chambers containing snails were rapidly transferred to a waterbath (Lab Companion RW-0525P, Korea). Snails were allowed to extract oxygen from the chamber water for precisely determined periods of ∼20 min, ensuring a reduction in the chamber O_2_ concentration that was detectable, but did not fall below 65% Air Saturation. In the *after* respiratory rate determinations, snails were given a shorter time to recover in seawater (10 min) in order to avoid missing a possible oxygen debt repayment (see Proum et al., 2017). The oxygen uptake incubation temperature for the air treatment was 27 ± 0.1°C and that for hypoxia seawater treatment was 30 ± 0.1°C; the latter was tweaked retrospectively to achieve a greater O_2_ extraction rate from the chamber water.

Oxygen concentration (% Air Saturation) was measured using a Witrox system (Loligo Systems, Denmark), comprising optical spot sensors glued inside each individual respiratory chamber and fiber-optic cables adhered to the outside of the chambers. After incubation, the snails were removed from their chambers and the oxygen concentration of the stirred water (magnetic bead) was logged every second until the readings stabilized (∼2 min) at temperatures of 26–27°C. For each set of measurements, oxygen concentrations were also determined for two control chambers lacking snails. Snail oxygen uptake was determined from the difference between the oxygen concentration in a chamber containing a snail and that of the average for the control chambers. Percentage air saturation values were converted to μmol L^–1^ O_2_ and then to μmol O_2_ h^–1^ g^–1^ wet tissue mass, after cracking open the shells and removing and weighing the tissue. The chamber water volume excluded the volume of the snail. Mass-specific *before* and *after* respiratory rates were compared with dependent *t*-tests, using Statistica v.12. All data exhibited homogeneity of variances (Levene’s test). Because of the narrow mass range of the snails used in each experiment, linear relationships for oxygen uptake versus mass were not significant (*p* = 0.78–0.89), precluding mass-scaling of respiratory rates. Mean snail wet masses (± SD) were 0.89 ± 0.2 g in air, 0.78 ± 0.16 g in hypoxia treatment 1, and 1.78 ± 0.1 g in hypoxia treatment 2.

In a separate experiment, we determined oxygen uptake during air (16 h) and hypoxia exposure (treatment 2, 16 h) for individual snails by measuring the oxygen concentration in closed chambers. Mass-specific oxygen uptake rates were extremely low in either medium; those in air (mean ± SD, 0.046 ± 0.008 μmol O_2_ h^–1^ g^–1^, wet mass = 1.26 ± 0.196 g, *n* = 6) were ∼3 times greater than those in hypoxic seawater (0.014 ± 0.004 μmol O_2_ h^–1^ g^–1^, wet mass = 1.79 ± 0.103 g, *n* = 4).

### Thermal Performance

#### Survival and Cardiac Performance During Acute Heat Exposure

Cardiac activity was monitored in snails that were heated in air (*n* = 7) or water (*n* = 10) from 30°C at 1°C for 10 min^–1^. Snails, previously fitted with IR-sensors, were retained in plastic bags (air) or 50 mL beakers containing aerated seawater (33 psu), inside a programmable temperature bath (Grant TXF200, Cambridge, United Kingdom). Heating started after 10 min equilibration at 29–30°C. Body temperature was determined from calibrated K-type thermocouples held next to the snails and connected to a TC-08 Picolog interface (Pico Technology, Cambridge, United Kingdom). Temperature and HR, measured in beats per minute (BPM), were logged simultaneously every 1 min. The HRs of individual snails were then averaged for every 1°C, and these values were used to determine mean HR for all snails for each temperature. The effect of temperature on HR for snails in air or water was determined using Friedman ANOVAs for multiple dependent samples (Statistica v.12, StatSoft, New York, United States).

Survival of acute heating was determined for snails (*n* = 17) heated in water from 30°C, after 10 min equilibration, to either 39, 41, 43, or 45°C at a rate of 1°C for 10 min^–1^, a rate that ensured thermal equilibration and physiological adjustment to each 1°C rise. Survival was ascertained by the re-emergence of snails at the shell aperture in air (24°C; see above), immediately after the heat treatment, and again after 24 h recovery in aquaria at 27°C. In cases where snails did not re-emerge, shells were cracked open to confirm mortality.

#### Survival of Chronic Heat Exposure

Although *T. bacillum* snails likely naturally experience only slight temperature variation (either daily or seasonally) during their lifetimes, acute heating experiments nonetheless inform about physiological capacity limitation, and thus are useful in comparisons between species (Marshall et al., 2011; Monaco et al., 2017). Acute heating protocols, irrespective of the habitat thermal regime, are also widely used to determine *thermal safety margins* and *warming tolerance* in studies assessing the climate vulnerability of animals (see Deutsch et al., 2008; Sinclair et al., 2016; Pinsky et al., 2019). To improve our understanding of the ability of these snails to tolerate temperatures experienced naturally, we assessed chronic heat exposure responses. Survival was determined for snails (*n* = 84) in air or water, kept at constant temperatures of 27, 33, 36, 38, and 40°C for 16 h (overnight). The number of snails surviving after each treatment was determined as above, immediately after the heat treatment, and again after 24 h recovery in seawater aquaria at 27°C.

## Results

### Oxygen Limitation Tolerance and Metabolic Depression

#### Survival and Cardiac Activity

**FIGURE 2:**
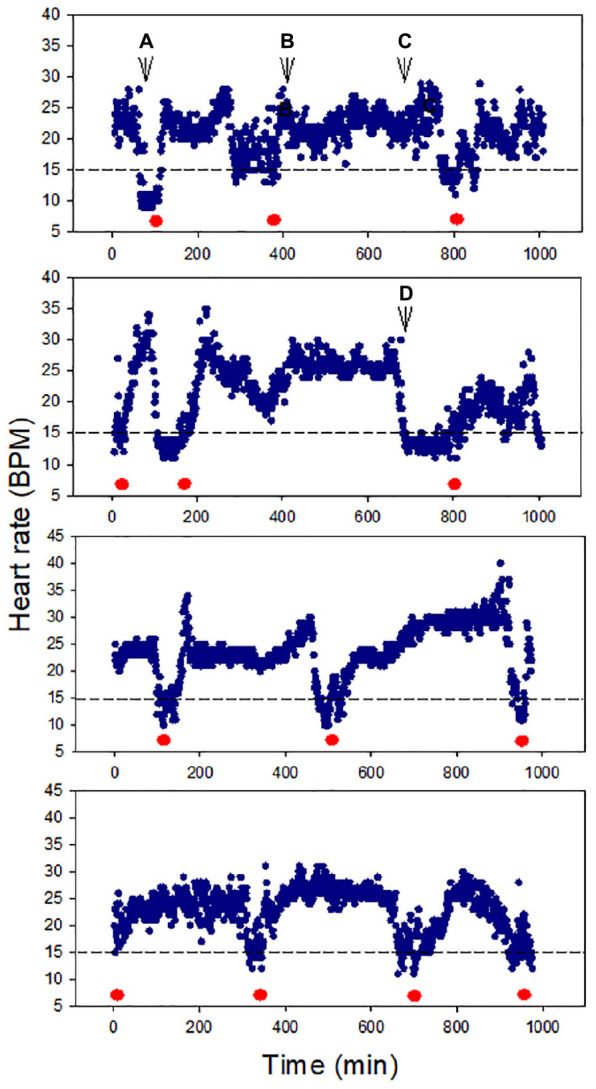
Heart rate (HR) modulation in four snails during 16 h air exposure (27 ± 0.2°C). All snails showed distinct cycles of depression and elevation of heart rate, including bradycardia (defined here as 15 BPM, red markers). Harmonic heart beating was observed throughout the 16 h period. Letters pinpoint where cardiograms shown in Figure 3 were collected.

**FIGURE 3:**
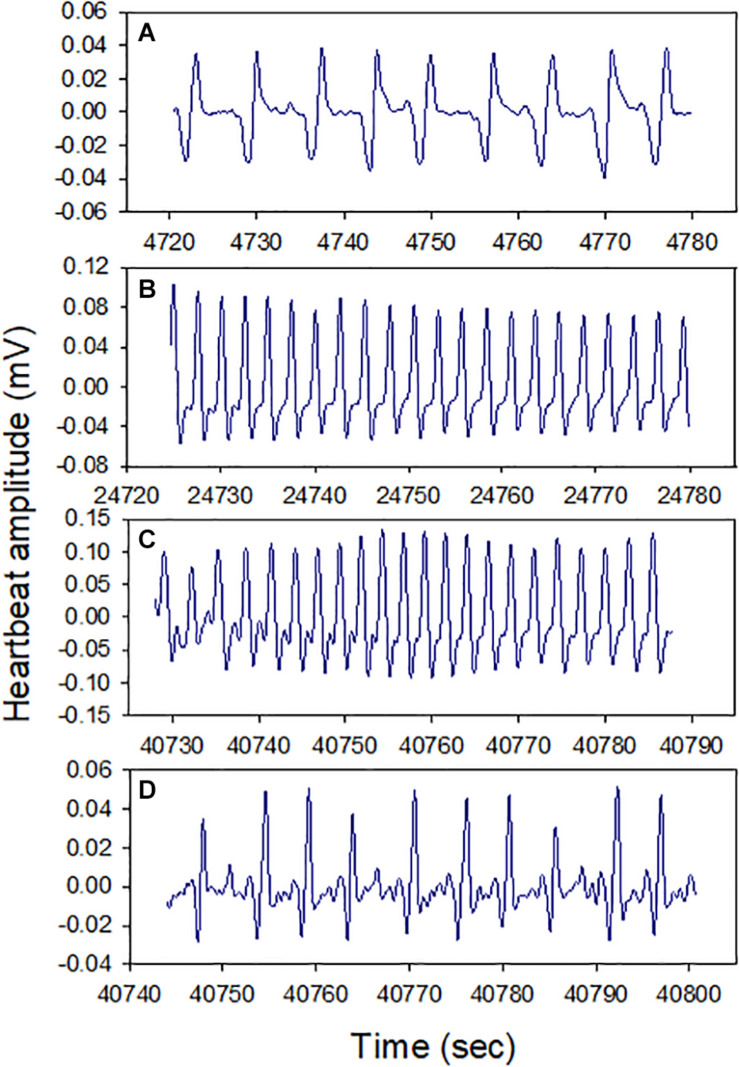
Cardiograms of snails at different times during 16 h air exposure (Letters **A–D** refer to points indicated in Figure 2). Heartbeat rhythmicity (beat to beat periodicity) was maintained throughout this exposure period in all snails.

All snails (100%) survived air exposure for up to 36 h, though 100% mortality occurred during 48 h exposure. A total of 100% survival was also observed after 16 h hypoxia exposure. In the cardiac activity experiments, all four snails showed HR recoverability during the 16 h air exposure period. The HR varied between ∼10 and 35 BPM, and there were clear episodes of recovery from bradycardia (<15 BPM), including after ∼13 h of air exposure (*p* = 0.0039; the probability of recovery occurring 3–4 times, compared to 0, 1, or 2 times, in each of four trails). The periodicity of bradycardia varied among individuals, with two showing evenly spaced episodes (Figure 2, bottom two panels). Heartbeat rhythmicity (beat–beat periodicity) was maintained for the entire exposure period, for faster or slower HRs (Figure 3). Similar heartbeat patterns were observed in the tracer snail exposed to hypoxic seawater. Bradycardia in this snail was characterized by sustained harmonic beating (Supplementary Figure 2).

#### Oxygen Debt Formation

**FIGURE 4:**
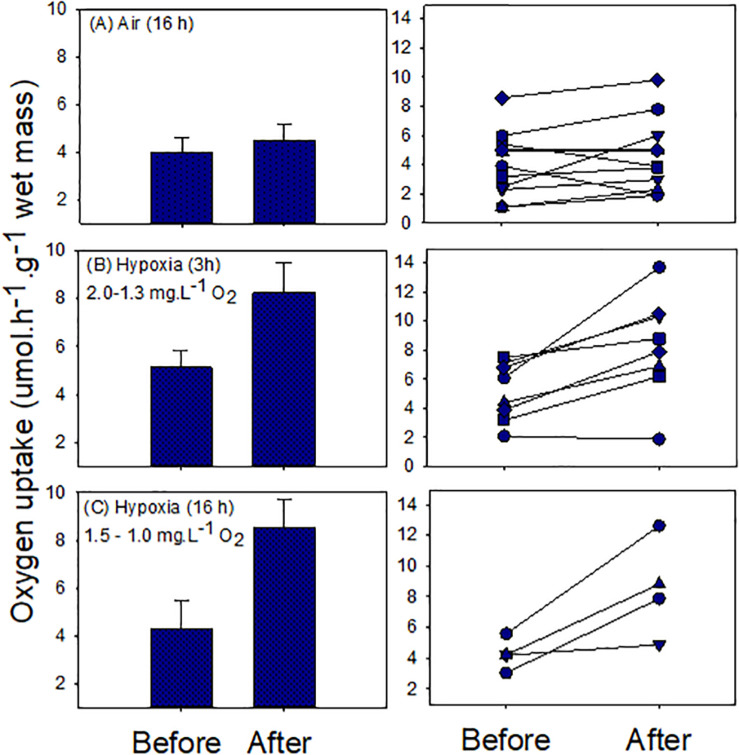
Oxygen uptake in normal seawater before and after exposure to air or hypoxic seawater. Left panels show means ± S.E. values and right panels show individual snail responses.

We found no evidence of oxygen debt formation in snails during air exposure (16 h). Although the mean after-exposure respiratory rate was greater than the before-exposure rate, the difference was not statistically significant [mean ± S.E.; 3.99 ± 0.63 and 4.5 ± 0.63 μmol h^–1^ g^–1^ wet mass (*t* = −1.22; *p* = 0.25; Figure 4]. Individual rates ranged between 1.1 and 8.6 and 1.9 and 9.8 μmol h^–1^ g^–1^ wet mass, for before- and after-exposure, respectively (Figure 4). In some cases the after-exposure respiratory rate increased whereas in others it decreased. Hypoxia exposure led to clear oxygen debt formation, with significant elevation of the mean after-exposure oxygen uptake rate. In experiment 1 (3 h), the mean values (± S.E.) were 5.19 ± 0.71 for before, and 8.27 ± 1.24 μmol h^–1^ g^–1^ wet mass, for after exposure (*t* = −3.91, *p* = 0.005; Figure 4). Individual rates varied between 2.1 and 7.5 for before and 1.9 and 13.7 μmol h^–1^ g^–1^ wet mass for after exposure. In only one case was the after-exposure rate not elevated (Figure 4). A similar result was obtained in experiment 2 (16 h). Mean values (± S.E.) were 4.28 ± 0.51 for before and 8.55 ± 1.59 μmol h^–1^ g^–1^ wet mass, for after exposure (*t* = −3.24, *p* = 0.048; Figure 4). Individual rates ranged between 3.1–5.5 and 4.1–12.6 μmol h^–1^ g^–1^ wet mass, respectively, for before- and after-exposure treatments.

### Thermal Performance and Tolerance

**FIGURE 5:**
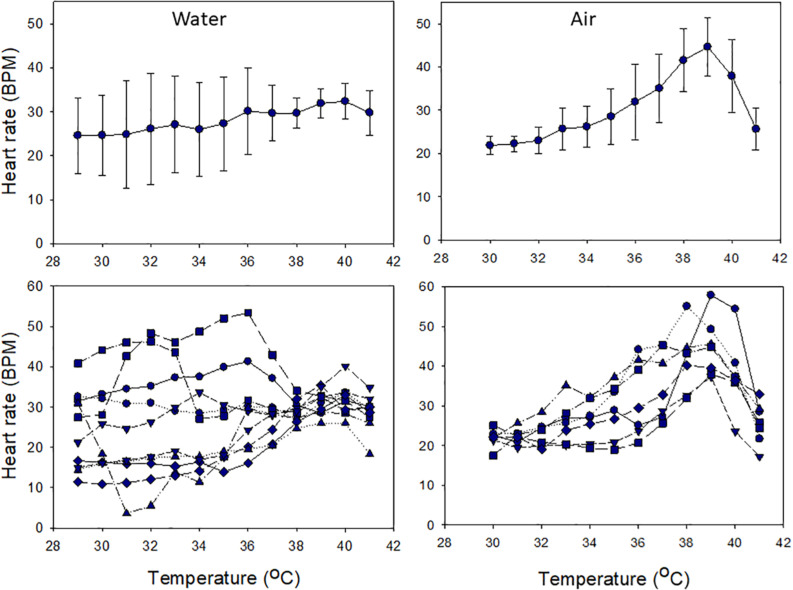
Heart rate responses of snails to heating in air or water from ∼29°C over 3 h. Upper panels show mean values (± S.D.), and lower panels show individual responses.

Cardiac performance under acute warming differed between snails in air and water. In water, mean HR was thermally insensitive, with little variation across the entire range of temperatures tested (24.1–31.6 BPM; 29–41°C; χ^2^ (df = 12) = 20.12, *p* = 0.065; Figure 5). Variation among individual HRs was high (averages ranged between 21.8 and 44.6 BPM), and HRs were largely thermally insensitive, but converged at ∼38°C (Figure 5). In contrast, HRs were temperature sensitive in air, increasing with heating up to 39°C and then decreasing [χ^2^ (df = 11) = 56.25, *p* < 0.001; Figure 5]. Flatlining occurred at or above 42°C. In air, mean HRs varied between 22 BPM at 30°C and 45 BPM at 39°C. Variability among individuals increased above 31°C, reflecting variability in their thermal sensitivities (note SD values and slopes in Figure 5).

Snails survived acute heat ramping in water up to 43°C (Table 2). The chronic thermal exposure experiments showed complete survival of snails in air or water up to a constant 36°C (16 h; Table 2). Whereas all snails survived exposure to 36°C when tested immediately after the experiment, none survived after 24 h (27°C, seawater) and no snails survived 40°C exposure (Table 2).

**TABLE 2:** Survival of heat exposure of *T. bacillum.*

Temp (°C)	Duration/medium	Survival% end (alive/total)	Survival% 24 h* (alive/total)
Acute**			
39	Ramping < 2 h/water	100 (4/4)	100 (5/5)
41	Ramping = 2 h/water	100 (5/5)	100 (5/5)
**43**	**Ramping > 2 h/water**	**100 (4/4)**	75 (3/4)
45	Ramping > 2 h/water	0 (0/4)	0 (0/4)
Chronic			
27	16 h/air	100 (12/12)	100 (12/12)
**36**	**16 h/air**	**75 (9/12)**	75 (9/12)
27	16 h/water	100 (12/12)	100 (12/12)
36	16 h/water	100 (12/12)	100 (12/12)
**38**	**16 h/water**	**100 (5/5)**	0 (0/5)
40	16 h/water	0 (0/5)	0 (0/5)

*Survival was assessed from snail responding to air exposure and the appearance of the operculum in aperture at the end of each thermal exposure and 24 h later (27°C, 33 ppt). **Ramp rate was 1°C for 10 min^–1^. Bold indicates highest temperature at which 50–100% of the population survived.

## Discussion

### Oxygen Limitation and Metabolic Depression

We show attributes of *T. bacillum* that indicate metabolic regulation rather than stress-related responses to oxygen uptake limitation. Metabolic downregulation is seen by (1) the suppression and recovery of HR, (2) rhythmic (harmonic) bradycardia, and (3) the absence of an oxygen debt when snails were exposed to air or hypoxic seawater. The responses of animals with little capability to regulate metabolism under oxygen limitation include a loss of heartbeat rhythmicity, long or continuous acardia, no recovery of HR, and an accumulation of an oxygen debt (Marshall and McQuaid, 1991, 1992a,b, 1993, 1994; Marshall et al., 2004; Proum et al., 2017). Although reduced cardiac activity often signals metabolic depression (Marshall and McQuaid, 1991, 1993, 2011; Marshall et al., 2004), we could not reconcile the HRs of *T. bacillum* snails with their low oxygen uptake rates in air or hypoxic seawater. Under these circumstances, the heart likely functions to maintain hemolymph circulation. In intertidal gastropods, HR relates to the rate of oxygen uptake in air and seawater when rates are varied by heating, but this relationship varies between the media due to impeded oxygen uptake in air-exposed snails (Marshall and McQuaid, 1993).

An oxygen debt reflects anaerobic metabolic compensation when aerobic ATP generation is reduced, and indicates an organism’s ability to downregulate overall metabolism (Hochachka, 1988; Grieshaber et al., 1994). A limited ability for downregulation corresponds with significant anaerobic compensation under declining cellular oxygen levels. The absence of or an inconsistent or weak oxygen debt formed in *T. bacillum* complies with observations of some intertidal gastropods (Brown and Wynberg, 1987; Marshall and McQuaid, 1993, 1994; Proum et al., 2017). Our data suggest a possible critical PO_2_ (the environmental O_2_ level at which ATP generation is largely anaerobic) for *T. bacillum* of between 2.2 and 1.5 mg L^–1^ O_2_. This was suggested by an hypoxia-induced oxygen-debt, and by the HR pattern of a tracer snail under hypoxia that recovered from bradycardia at 2.2 mg L^–1^ O_2_ seawater, but not at lower oxygen concentrations (<1.5 mg L^–1^ O_2_). Striking individual differences were seen in oxygen debt formation during hypoxia, implying different energetic requirements of individual snails. Some individuals may need to complete energy-demanding, intrisically fixed stages of digestion, reproduction and growth (requiring anaerobic compensation), before reverting to an adaptive energy-conserving hypometabolic state (see Marshall et al., 2004; Marshall and McQuaid, 2011).

Metabolic regulation characterizes land-dwelling and intertidal gastropods, but is poorly reported in subtidal gastropods, despite their often exceptional tolerance of hypoxia (Stickle et al., 1989; Liu et al., 1990; Guppy and Withers, 1999; Sokolova and Pörtner, 2001, 2003; Storey, 2002; Marshall and McQuaid, 2011; Marshall et al., 2011; Riedel et al., 2012, 2014). By facilitating tolerance of environmental stress and food shortages, regulation underpins the exceptional evolutionary transitions and radiations by gastropods (Webb, 2012). A hypometabolic capability should benefit *T. bacillum* by allowing it to withstand restrictions on food and oxygen supply to the cells. Environmental hypoxia occurs widely in oceanic environments, especially in association with upwelling, and is expected to worsen as coastal eutrophication and global warming progress (Vaquer-Sunyer and Duarte, 2008; Riedel et al., 2012). Hypoxic conditions can also be induced behaviorally in sediment-dwelling and burrowing marine gastropods (Brown and Da Silva, 1979; Marshall, 2009; Proum et al., 2017). *T. bacillum* snails survived in the laboratory for weeks without food or when completely buried with no contact with the above-sediment water (see section “Materials and Methods”). These snails should experience natural disruptions in food supply when buried or when feeding is curtailed by high suspended sediment loads (Ellis et al., 2002; Lohrer et al., 2004). The observation of metabolic depression during air exposure, however, does not necessarily mean an evolutionary association with the intertidal ecosystem, but rather likely represents a general physiological response to reduced oxygen uptake (see below).

### Thermal Performance and Tolerance

Whereas tolerance of air exposure can be explained by the capacity of *T. bacillum* for hypometabolism and hypoxia tolerance, its heat tolerance and the difference between aerial and aquatic thermal performance were unexpected. Because oxygen uptake is not inhibited in normoxic seawater, we assume that the thermal insensitivity of cardiac activity in snails reflects temperature-independent metabolism (Marshall and McQuaid, 1993; Verberk et al., 2016), which corresponds with the thermal stability of the habitat of these snails (25–31°C; Supplementary Figure 1). However, because oxygen uptake is severely limited in air, the observed thermal dependence of aerial HR indicates uncoupling of cardiac and metabolic performance. Despite this lack of functional association, the aerial cardiac performance of *T. bacillum* was not random and was clearly maximized at 39°C. The high individual variability in HR and in thermal sensitivity again likely refers to different energy demands and stable states of individuals (see above). The initially variable HRs in water converged at 38°C, and variably sensitive individual rates in air-exposed snails peaked at 39°C. Together, these data suggest a physiological threshold, with optimal thermal performance (*T*_*opt*_) at 38–39°C. Flatlining of HR under acute heating indicates an upper thermal limit and the onset of mortality at ∼42°C (Stenseng et al., 2005; Polgar et al., 2015). This was confirmed by the highest temperature for 100% survival under acute ramping of 43°C.

It is difficult to ascribe adaptive significance to the observed thermal performance of *T. bacillum* given the habitat temperatures it naturally experiences. Its thermal physiology suggests trait conservation, with unexpressed phenotypes under natural circumstances being retained in evolutionary lineages (Angilletta, 2009; Grigg and Buckley, 2013). Although *T. bacillum* is thought to have originated since the Plio-Pleistocene period (Das et al., 2018), older turriteline lineages date to the Cretaceous and have survived the Paleocene-Eocene Thermal Maximum (see Ivany et al., 2018), the hottest time in the history of the living planet. Alternatively, heat tolerance may be a more recent acquisition, linked to earlier occupation of the thermally variable intertidal zone. The closest generic relative of *T. bacillum* is an intertidal snail (*Batillaria*; Strong et al., 2011) and its cardiac activity (in air) bears similarity to that of low-shore species, *Turbo bruneus* and *Trochus radiatus* (Monaco et al., 2017). Phylogenetic conservatism of thermal tolerance in niche-radiating gastropods is nicely exemplified by the gross mismatch between habitat temperature and thermal physiology in an equatorial freshwater snail (*Clea nigricans*), belonging to a predominantly marine and intertidal clade (Buccinoidea; Polgar et al., 2015). Disentangling these possible interpretations requires a solid trait-based phylogeny.

### Climate Vulnerability and Physiological Diversity

Our findings exemplify the difficulty of making general predictions for climate vulnerability in marine ectotherms. Such predictions are commonly founded on relationships between species’ thermal tolerances, habitat temperature conditions, and expected long-term environmental warming rates. For benthic animals these inferences are not straightforward, as species thermal limits can be flexible, can vary with experimental heating rates, and rates of ocean warming can vary spatially. Nonetheless, a *warming tolerance* metric calculated as the difference between a species’ upper thermal limit and maximum habitat temperature, provides some insight into the probability of species persistence. The broadscale application of this approach across latitudinal gradients suggests that tropical species are particularly vulnerable to a predicted 2–3°C increase in centennial temperature (Deutsch et al., 2008; Huey et al., 2009). In the case of *T. bacillum*, however, a *warming tolerance* of 7°C, based on our most conservative estimates of heat tolerance (100% survival for 16 h at 38°C) and mean maximum habitat temperature of 31°C (Supplementary Figure 1), far exceeds this general prediction. We recognize the importance of chronic heating to climate vulnerability in marine subtidal animals (Peck et al., 2009; Richard et al., 2012), but note that tolerance of *T. bacillum* of acute heat ramping (100% survival to 43°C) is greater than that recorded for other tropical subtidal gastropods (e.g., 41.1–42.4°C; Nguyen et al., 2011). We are also cognizant of the fact that past extinctions of turritelline snails are ascribed to changes in global temperature, suggesting warming vulnerability of the group in general, but not necessarily of specific cases (Anderson and Allmon, 2020).

The mechanisms underlying the persistence of subtidal marine populations and species over temperature changes spanning decades are complex and likely to relate to organismal energetics, rather than thermal tolerance limits. Current models based on ‘fixed’ energy metabolism of individuals imply that survival at higher temperatures requires an increase in energy intake (food) to meet thermally elevated energy demands (Brown et al., 2004; Van der Meer, 2006; Sinclair et al., 2016; Huey and Kingsolver, 2019). Such elevated energy demands occur at the same time that performance becomes suboptimal and food availability is likely limited by reduced production and generally a reduced energy flow through the ecosystem (see Morais et al., 2020). However, these energetic constraints can be negated or minimized in animals with flexible or temperature independent metabolic rates that allow the lowering of resting energy demands, as occurs in many gastropods, including *T. bacillum*.

Accounting for metabolic regulation where this physiology exists, rather than assuming “fixed” metabolism, is crucial to predicting responses to climate warming. The capacity for metabolic regulation appears to exist in marine “pulmonates” and caenogastropod groups (Buccinoidea, Littorinoidea, Muricoidea, and Cerithioidea), while seems less well developed in the Patellogastropoda and the Vetigastropoda (McMahon and Russell-Hunter, 1977; Kapper and Stickle, 1987; Liu et al., 1990; Marshall and McQuaid, 1991, 1992a,b, 1993, 1994; Riedel et al., 2012; Leung et al., 2017). *T. bacillum* belongs to the Cerithoidea, a group that has radiated in physically variable and extreme fringe environments, including rocky-shores, mangroves, estuaries and brackwater streams. Although no phylogenetic data are available to confirm precedence of hypometabolic physiology in subtidal cerithioideans (Strong et al., 2011), exaptation of this trait in fringe lineages is suggested by the antiquity of turritelline snails (Marshall and McQuaid, 1991).

## Conclusion

*Turritella bacillum* exhibited physiological features consistent with metabolic regulation and heat tolerance. Metabolic depression sustains environmental isolation, prolonged starvation and tolerance of hypoxia, which enable animals to withstand environmental deterioration, and which should provide resilience against climatic-driven environmental change. Though common in marine gastropods, hypometabolism is poorly integrated into contemporary hypotheses concerning responses to climate change, which center on the optimization of energy intake and “fixed” metabolic rates (Van der Meer, 2006). The general hypothesis that tropical marine animals are especially vulnerable to climate warming (Deutsch et al., 2008; Tewksbury et al., 2008; Sunday et al., 2014; Pinsky et al., 2019) is not supported by our findings. Attempts to predict species responses to climate change would benefit from considering greater physiological diversity (Somero, 2010), rather than focusing on increasing the count of physiologically similar species. Identifying and including information for taxa that show metabolic regulatory physiology should enhance the accuracy of climate vulnerability predictions.

## Data Availability Statement

The datasets generated for this study are available on request to the corresponding author.

## Author Contributions

DM performed the research. DM and CM formulated the concept and wrote the manuscript. All authors contributed to the article and approved the submitted version.

## Conflict of Interest

The authors declare that the research was conducted in the absence of any commercial or financial relationships that could be construed as a potential conflict of interest.
