# Supplementary material for: Metabolic Regulation, Oxygen Limitation and Heat Tolerance in a Subtidal Marine Gastropod Reveal the Complexity of Predicting Climate Change Vulnerability
Source: Front Physiol. 2020 Sep 15;11:1106. doi: 10.3389/fphys.2020.01106 (PMC7556210; doi:10.3389/fphys.2020.01106)
Supplement: Supplementary file 1 [file Image_1.pdf]

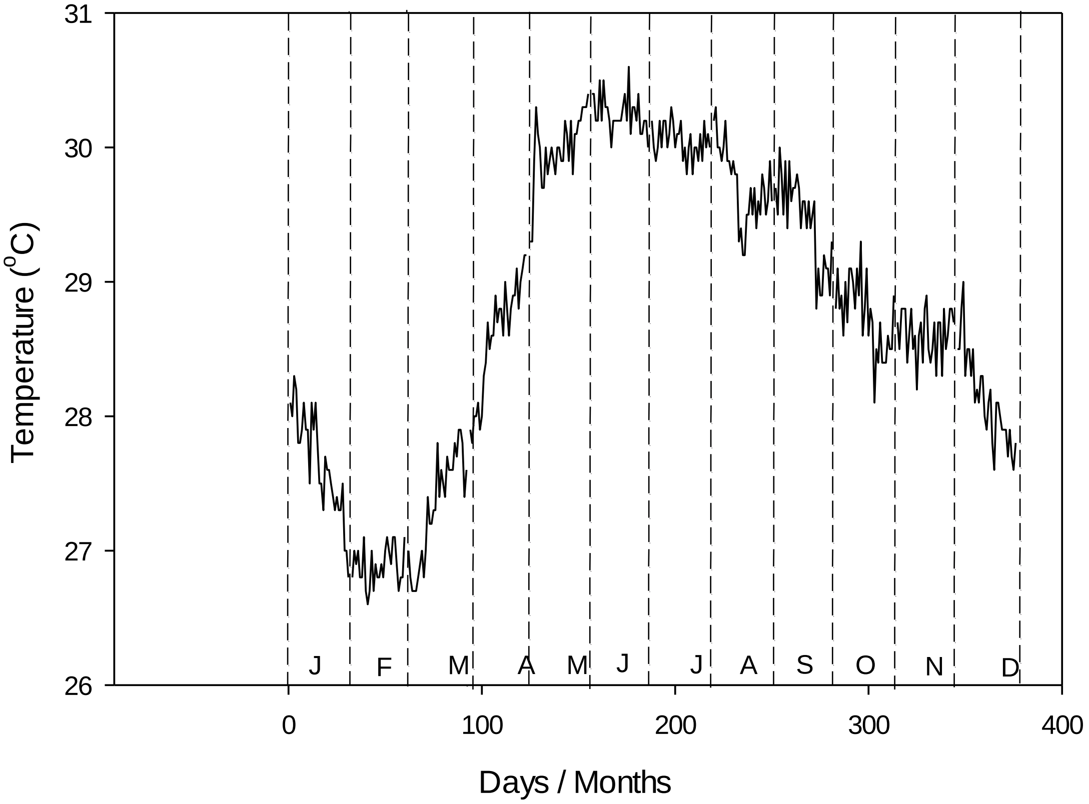

Supplementary Figure 1. Daily seawater temperature in Brunei (2019) (Brunei Weather Bureau). Published studies suggest a 25-29.5°C range recorded at 50 m depth off Brunei (Johari and Akhir, 2019) and 25.2 – 30.5°C (11 m) range, at inshore coral reefs (Lane, 2011)
