# Supplementary material for: Metabolic Regulation, Oxygen Limitation and Heat Tolerance in a Subtidal Marine Gastropod Reveal the Complexity of Predicting Climate Change Vulnerability
Source: Front Physiol. 2020 Sep 15;11:1106. doi: 10.3389/fphys.2020.01106 (PMC7556210; doi:10.3389/fphys.2020.01106)
Supplement: Supplementary file 2 [file Image_2.pdf]

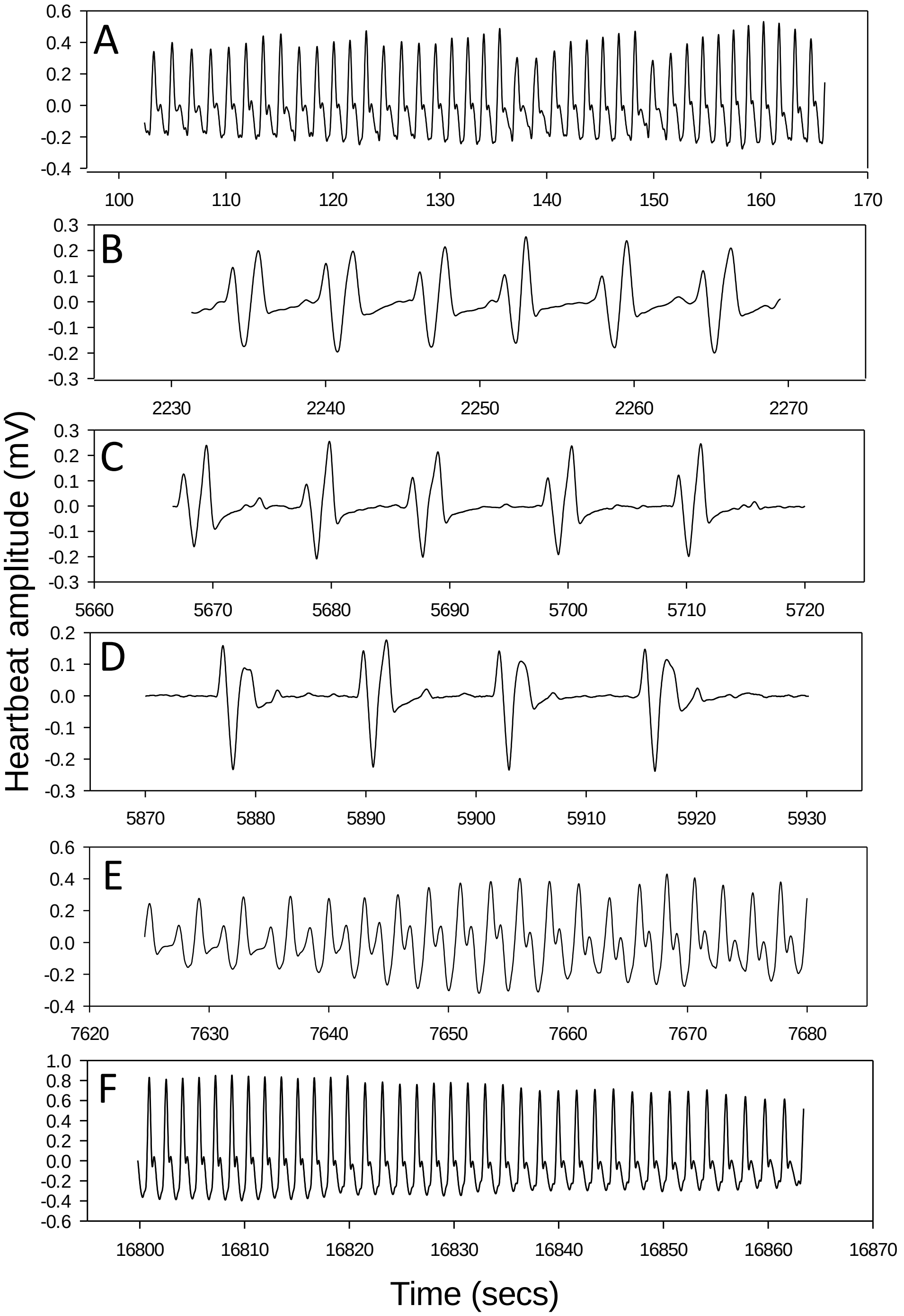
A
B
C
D
E
F
Heartbeat amplitude (mV)
Time (secs)

Supplementary Figure 2. Cardiograms of a single snail before, during and after exposure to hypoxic seawater. (A), (B), (E) and (F), normoxia; (C), hypoxia 2.3 $mg.L^{-1}$; (D), hypoxia 1.5 $mg.L^{-1}$. Heart beat periodicity was maintained throughout the experiment, including during bradycardia. Bradycardia was induced during initial normoxia, and heart beat recovered during hypoxia at O2 concentrations of 2.3 $mg.L^{-1}$, but not during 1.5 $mg.L^{-1}$ O_2 concentration. Note the relatively high amplitude in (F) is consistent with a larger stroke volume and repayment of O_2-debt.
